# Early identification of delayed extubation following cardiac surgery: Development and validation of a risk prediction model

**DOI:** 10.3389/fcvm.2022.1002768

**Published:** 2022-10-04

**Authors:** Xia Li, Jie Liu, Zhenzhen Xu, Yanting Wang, Lu Chen, Yunxiao Bai, Wanli Xie, Qingping Wu

**Affiliations:** Department of Anesthesiology, Union Hospital, Tongji Medical College, Huazhong University of Science and Technology, Wuhan, China

**Keywords:** delayed extubation, risk factor, cardiac surgery, risk score, prediction model

## Abstract

**Background:**

Successful weaning and extubation after cardiac surgery is an important step of postoperative recovery. Delayed extubation is associated with poor prognosis and high mortality, thereby contributing to a substantial economic burden. The aim of this study was to develop and validate a prediction model estimate the risk of delayed extubation after cardiac surgery based on perioperative risk factors.

**Methods:**

We performed a retrospective cohort study of adult patients undergoing cardiac surgery from 2014 to 2019. Eligible participants were randomly assigned into the development and validation cohorts, with a ratio of 7:3. Variables were selected using least absolute shrinkage and selection operator (LASSO) logistic regression model with 10-fold cross-validation. Multivariable logistic regression was applied to develop a predictive model by introducing the predictors selected from the LASSO regression. Receiver operating characteristic (ROC) curve, calibration plot, decision curve analysis (DCA) and clinical impact curve were used to evaluate the performance of the predictive risk score model.

**Results:**

Among the 3,919 adults included in our study, 533 patients (13.6%) experienced delayed extubation. The median ventilation time was 68 h in the group with delayed extubation and 21 h in the group without delayed extubation. A predictive scoring system was derived based on 10 identified risk factors based on 10 identified risk factors including age, BMI ≥ 28 kg/m^2^, EF < 50%, history of cardiac surgery, type of operation, emergency surgery, CPB ≥ 120 min, duration of surgery, IABP and eGFR < 60 mL/min/1.73 m^2^. According to the scoring system, the patients were classified into three risk intervals: low, medium and high risk. The model performed well in the validation set with AUC of 0.782 and a non-significant *p*-value of 0.901 in the Hosmer-Lemeshow test. The DCA curve and clinical impact curve showed a good clinical utility of this model.

**Conclusions:**

We developed and validated a prediction score model to predict the risk of delayed extubation after cardiac surgery, which may help identify high-risk patients to target with potential preventive measures.

## Introduction

With innovations and advances in medical management and surgical techniques, a growing number of patients undergo cardiac surgery, including critically ill patients with multiple comorbidities (1). Although most of the patients can resume spontaneous respiration as soon as possible after cardiac surgery, successful weaning from mechanical ventilation remains a challenge (2, 3). Due to prolonged ventilator support, up to 20% of patients experienced delayed extubation after cardiac surgery, which increased the risk of postoperative complications including acute kidney injury and delirium (4, 5). More notably, among patients requiring prolonged mechanical ventilation, the recently observed in-hospital mortality rate was as high as 50.3% (6, 7). Additionally, the need for prolonged postoperative mechanical ventilation imposes a burden on the allocation of critical care resources such as ventilator and intensive care unit (ICU) beds. Therefore, early identification of patients at risk of delayed extubation appears important.

A comprehensive assessment system incorporating preoperative and intraoperative factors can accurately predict the risk of delayed extubation after cardiac surgery and guide clinical decision-making. Although some predictive scoring systems for delayed extubation after cardiac surgery have been reported (8–11), specifically, sample size issues, single type of surgery and imperfection of assessment system in these studies have limited the application and promotion of the predictive models in clinical practice. Moreover, the majority of these participant were from developed countries. Considering the differences in lifestyle, income and medical resources, a novel predictive model that can be widely used in developing countries is still needed.

The risk factors for delayed extubation are mainly attributed to patient-related and surgery-related factors, but the optimal combination of predictors has not been identified. Therefore, the purpose of this study was first to evaluate the duration of postoperative mechanical ventilation and then identify the potential risk factors for delayed extubation. Ultimately, the results were used to establish and validate a model to help clinicians understand the likelihood of delayed extubation and allocate postoperative medical resources appropriately at the first time after cardiac surgery.

## Methods

### Ethical statement

Permission for this retrospective study was obtained from the Ethics Committee of Union Hospital affiliated to Tongji Medical College of Huazhong University of Science and Technology (No. 2022-0465). The written informed consent was waived by the institutional review boards because of the retrospective and anonymous nature of the data. This study was conducted in accordance with the ethical principles stated in the Declaration of Helsinki and reported according to TRIPOD guidelines (Supplementary Table 1) (12).

### Study design and population

From January 2014 to December 2019, all consecutive adult patients undergoing cardiac surgery were enrolled in this retrospective observational study at Union Hospital in Wuhan, China. Surgical procedures were extensive, including coronary artery bypass graft (CABG), valve repair or replacement procedures (single or multiple), aortic surgery (due to aortic aneurysm and dissection) and combined surgeries. Because of the retrospective and observational nature, no formal sample size calculation was performed.

Participants were excluded from this study if they met any of the following criteria: (1) preoperative mechanical ventilation; (2) combined with non-cardiac surgeries like lobectomy; (3) second thoracotomy after surgery; (4) died or discharged within 48 h after surgery; (5) clinical data loss.

### Data collection

Demographic and clinical information were extracted from the electronic medical record of the hospital. Preoperative variables included age, gender, body mass index (BMI), history of smoking and alcohol, diabetes, hypertension, lung disease, heart disease, ejection fraction (EF), cerebrovascular disease, cancer history, renal insufficiency, surgery history and use of vasoactive agents and antibiotics. Pulmonary arterial hypertension (PAH) was defined Doppler echocardiography or other results of the hemodynamic tests. Cerebrovascular disease included any history of stroke, transient ischemic attack, carotid endarterectomy, and carotid stenting. Preoperative medication was defined as 1 week prior to surgery and used for more than 3 consecutive days.

Intraoperative factors were as follows: type of surgery classified in six categories, emergency surgery, surgical approach (median sternotomy vs. minimally invasive), cardiopulmonary bypass (CPB) and aortic cross clamp (ACC) time, transfusion of blood product, operative duration and use of intra-aortic balloon pump (IABP). Autologous transfusion was excluded from the transfusion record.

Preoperative laboratory test results included neutrophil–lymphocyte ratio (NLR), platelet–lymphocyte ratio (PLR), lymphocyte-to-monocyte ratio (LMR), hemoglobin, albumin, estimated glomerular filtration rate (eGFR). Anemia was defined as hemoglobin < 130 g/L for men or < 120 g/L for women.

### Study outcomes

Delayed extubation was defined as postoperative mechanical ventilation lasting >48 h or reintubation within the first 2 days after surgery. Also, other possible outcomes were recorded: length of ICU stay and postoperative hospital days, major postoperative complications and in-hospital mortality.

Neurological complications included cerebral ischemic infarct or intracerebral hemorrhage with the presence of imaging evidence. Pneumonia and cardiac arrest were defined according to European Perioperative Clinical Outcome (EPCO) definitions (13).

### Statistical analysis

In the initial variable pool, two variables that were missing in >20% of patients were directly removed, which were forced expiratory volume in 1 s (FEV_1_) to forced expiratory volume (FCV) ratio and New York Heart Association (NYHA) classification. Then, we again excluded individuals with missing variables, including those with missing rate < 5%. As such, no statistical interpolation techniques were used in our dataset to ensure high clinical relevance. According to clinical relevance and practicality, we transformed the corresponding continuous variables into categorical variables. Data were not normally distributed. For demographic and clinical variables, continuous variables were described using medians with interquartile ranges (IQR), and categorical variables were summarized as frequencies with percentage. Differences between groups were compared using the chi-square tests for categorical data and nonparametric tests for continuous data as appropriate.

The full cohort was randomly assigned into the development and validation sets at a 7:3 ratio. We used the training cohort to derive a risk prediction score system and then externally validated the score system in the validation cohort. Least absolute shrinkage and selection operator (LASSO) regression is a very popular technique to shrink and select variables for regression models (14). First, we use LASSO regression to narrow the scope of the candidate predictors, using cross-validation as the criterion to select variables. The variables selected in the LASSO analysis were entered into multivariable logistic regression analysis. Then, variables with *P*-value < 0.05 were applied to construct a risk prediction score system for delayed extubation after cardiac surgery. In order to facilitate visualization, predicted risk scores were assigned by dividing the regression coefficient of each significant factor by the smallest β coefficient and then rounded to the closest integer. The total point for each patient was calculated by summing the scores of existing risk factors. Patients in the cohort were ranked and classified into 3 risk groups based on the tertiles of total risk scores: low risk (< 33%), medium risk (33–66%), and high risk >66%.

The risk prediction model was evaluated and validated using the data of training set and validation set, respectively. Receiver operating characteristic (ROC) curve was used to access the capability of the predictive model to discriminate true positives from false positives. The accuracy of model was evaluated by Hosmer-Lemeshow goodness-of-fit test and calibration curve. Clinical usefulness and net benefit of the model were estimated with decision curve analysis (DCA) and clinical impact curve. All statistical analysis was performed using R software (version 4.2.0). A *P*-value of < 0.05 was considered statistically significant.

## Results

### Characteristics of the study cohort

**Figure 1:**
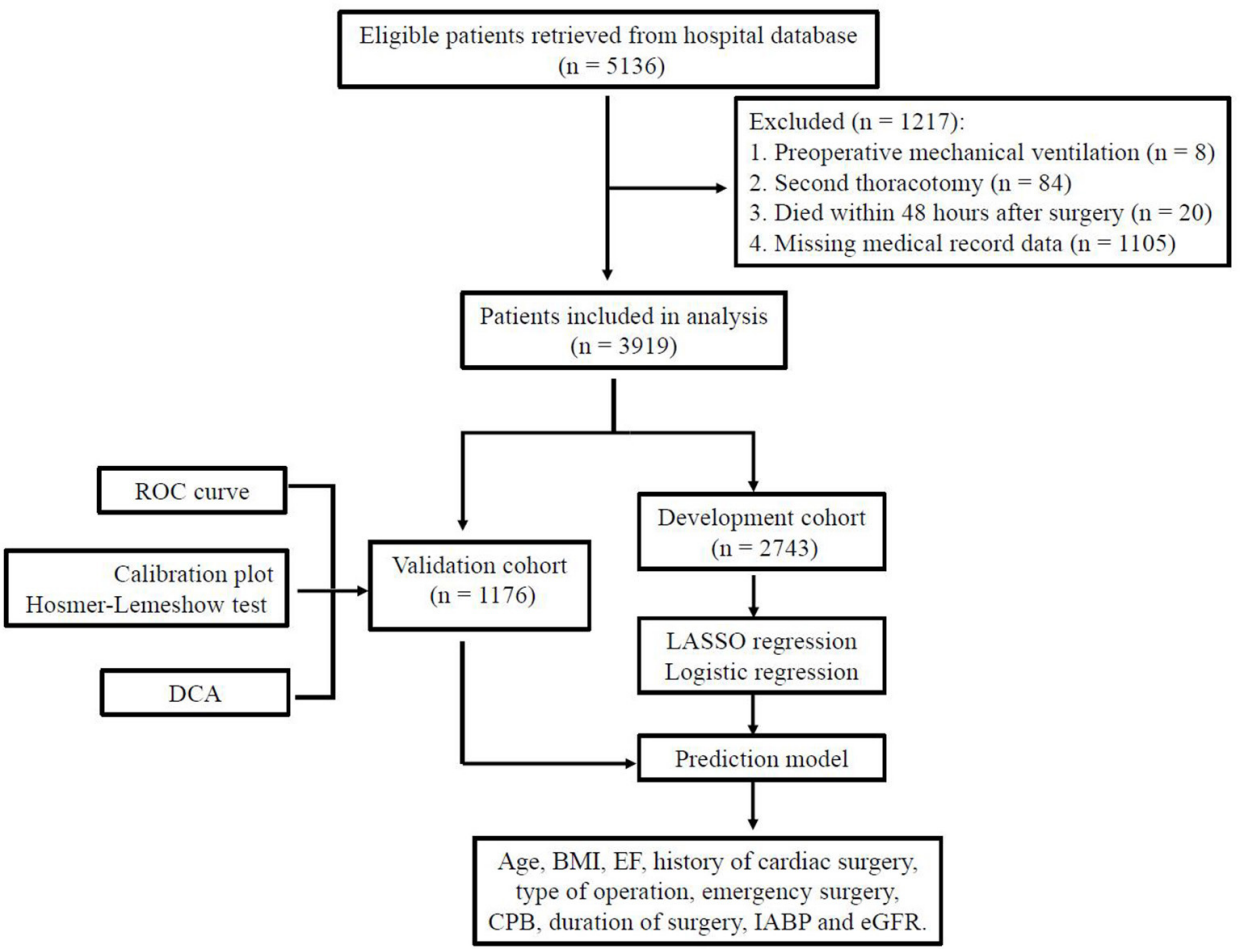
The flowchart of study design and data analysis.

Data of 5,136 consecutive cases were retrieved from the hospital record database and 1,217 patients were excluded (Figure 1). A total of 3,919 patients were included in this study: 777 (19.8%) isolated coronary artery bypass grafting (CABG), 2,452 (62.6%) isolated valve surgery, 238 (6.1%) isolated aortic surgery, 380 (9.7%) combined CABG and valve procedures, 37 (0.9%) combined valve and aortic procedures and 35 (0.9%) combined CABG and aortic procedures. Of the 3,919 participants in our analysis, 533 (13.6%) patients experienced delayed extubation. Compared with patients without delayed extubation, patients with delayed extubation were older and had a higher percentage of comorbidities such as hypertension, coronary heart disease, atrial fibrillation, previous myocardial infarction, pulmonary disease, cerebrovascular disease and renal insufficiency (Table 1). All of the 3,919 patients were randomly assigned into two independent sets: the training cohort (*n* = 2,743) and the validation cohort (*n* = 1,176). We compared details of the demographic and clinical variables between these two groups and results show that there were no significant differences in demographic characteristics (Table 1). In summary, the training cohort and the validation cohort shared similar characteristics, including the incidence of delayed extubation (13.7 vs. 13.3%, *P* = 0.689).

**Table 1:** The demographic and clinical characteristics of patients in the training and validation datasets.

**Variables**	**Total** **(*n* = 3,919)**	**Delayed extubation (*n* = 533)**	**Without delayed extubation** **(*n* = 3,386)**	***P*-value**	**Training set** **(*n* = 2,743)**	**Validation set** **(*n* = 1,176)**	***P*-value**
**Pre-operation**							
Age (years)	54 (46, 62)	58 (50,65)	54 (46, 62)	< 0.001	54 (46, 62)	54 (46,62)	0.293
Age (years)				< 0.001			0.427
< 40	582 (14.9)	42 (7.9)	540 (15.9)		408 (14.9)	174 (14.8)	
40–50	728 (18.6)	86 (16.1)	642 (19.0)		499 (18.2)	229 (19.5)	
50–60	1,256 (32.0)	162 (30.4)	1,094 (32.3)		868 (31.6)	388 (33.0)	
60–70	1,130 (28.8)	193 (36.2)	937 (27.7)		815 (29.7)	315 (26.8)	
≥70	223 (5.7)	50 (9.4)	173 (5.1)		153 (5.6)	70 (6.0)	
Male	2,273 (58.0)	348 (65.3)	1,925 (56.9)	< 0.001	1,610 (58.7)	663 (56.4)	0.178
BMI (kg/m^2^)	23.4 (21.3,25.7)	23.6 (21.3,26.1)	23.4 (21.3,25.6)	0.192	23.4 (21.3,25.7)	23.4 (21.3,25.7)	0.839
BMI ≥ 28 kg/m^2^	357 (9.1)	76 (14.5)	281 (8.3)	< 0.001	253 (9.2)	104 (8.8)	0.705
Smoking history	1,206 (30.8)	198 (37.1)	1,008 (29.8)	0.001	863 (31.5)	343 (29.2)	0.154
Drinking history	870 (22.2)	141 (26.5)	729 (21.5)	0.011	628 (22.9)	242 (20.6)	0.110
Hypertension	1,243 (31.7)	221 (41.5)	1,022 (30.2)	< 0.001	881 (32.1)	362 (30.8)	0.410
Diabetes	452 (11.5)	67 (12.6)	385 (11.4)	0.420	321 (11.7)	131 (11.1)	0.613
COPD	60 (1.5)	11 (2.1)	49 (1.4)	0.281	40 (1.5)	20 (1.7)	0.571
Recent pneumonia	165 (4.2)	34 (6.4)	131 (3.9)	0.007	110 (4.0)	55 (4.7)	0.341
CAD	1,380 (35.2)	223 (41.8)	1,157 (34.2)	0.001	977 (35.6)	403 (34.3)	0.418
History of MI	174 (4.4)	38 (7.1)	136 (4.0)	0.001	120 (4.4)	54 (4.6)	0.762
History of PCI	115 (2.9)	18 (3.4)	97 (2.9)	0.515	79 (2.9)	36 (3.1)	0.758
PAH	183 (4.7)	30 (5.6)	153 (4.5)	0.259	134 (4.9)	49 (4.2)	0.329
Atrial fibrillation	694 (17.7)	113 (21.2)	581 (17.2)	0.023	484 (17.6)	210 (17.9)	0.873
EF (%)	62 (58,66)	60 (53, 65)	62 (58, 66)	< 0.001	61 (57, 65)	62 (58, 66)	0.146
EF < 50%	313 (8.0)	101 (18.9)	212 (6.3)	< 0.001	224 (8.2)	89 (7.6)	0.527
Cerebrovascular disease	230 (5.9)	50 (9.4)	180 (5.3)	< 0.001	158 (5.8)	72 (6.1)	0.658
Renal insufficiency	144 (3.7)	36 (6.8)	108 (3.2)	< 0.001	97 (3.5)	47 (4.0)	0.483
Cancer history	24 (0.6)	5 (0.9)	19 (0.6)	0.300	18 (0.7)	6 (0.5)	0.591
Prior cardiac surgery	161 (4.1)	43 (8.1)	118 (3.5)	< 0.001	113 (4.1)	48 (4.1)	0.956
History of abdominal or thoracic surgery	175 (4.5)	29 (5.4)	146 (4.3)	0.241	116 (4.2)	59 (5.0)	0.274
Vasoactive drugs	92 (2.3)	35 (6.6)	57 (1.7)	< 0.001	70 (2.6)	22 (1.9)	0.197
Antibiotic use	594 (15.2)	101 (18.9)	493 (14.6)	0.009	424 (15.5)	170 (14.5)	0.423
**Intra-operation**							
Type of operation				< 0.001			0.817
CABG only	777 (19.8)	85 (15.9)	692 (20.4)		542 (19.8)	235 (20.0)	
Valve only	2,452 (62.6)	237 (44.5)	2,215 (65.4)		1,713 (62.4)	739 (62.8)	
Aortic only	238 (6.1)	74 (13.9)	164 (4.8)		165 (6.0)	73 (6.2)	
CABG+ valve	380 (9.7)	107 (20.1)	273 (8.1)		275 (10.0)	105 (8.9)	
Valve + aortic	37 (0.9)	12 (2.3)	25 (0.7)		23 (0.8)	14 (1.2)	
CABG+ aortic	35 (0.9)	18 (3.4)	17 (0.5)		25 (0.9)	10 (0.9)	
Emergency surgery	71 (1.8)	41 (7.7)	30 (0.9)	< 0.001	47 (1.7)	24 (2.0)	0.481
Surgical incision				< 0.001			0.640
Median sternotomy	3,539 (90.3)	512 (96.1)	3,027 (89.4)		2,481 (90.4)	1,058 (90.0)	
Minimally invasive	380 (9.7)	21 (3.9)	359 (10.6)		262 (9.6)	118 (10.0)	
Surgery time (h)	4.0 (3.3, 4.9)	4.8 (4.0, 6.0)	4.0 (3.2, 4.7)	< 0.001	4.0 (3.3, 5.0)	4.0 (3.3, 4.8)	0.163
Duration of surgery				< 0.001			0.140
< 3 h	414 (10.6)	19 (3.6)	395 (11.7)		292 (10.6)	122 (10.4)	
3–5 h	2,552 (65.1)	261 (49.0)	2,291 (67.7)		1,758 (64.1)	794 (67.5)	
5–7 h	742 (18.9)	150 (28.1)	592 (17.5)		544 (19.8)	198 (16.8)	
≥7 h	211 (5.4)	103 (19.3)	108 (3.2)		149 (5.4)	62 (5.3)	
CPB time (min)	102 (74,134)	130 (97,177)	98 (72,128)	< 0.001	102 (74, 134)	101 (73,132)	0.367
CPB ≥ 120 min	1,376 (35.1)	322 (60.4)	1,054 (31.1)	< 0.001	972 (35.4)	404 (34.4)	0.515
ACC time (min)	67 (44, 90)	85 (59, 114)	65 (42, 87)	< 0.001	67 (44, 90)	67 (43, 91)	0.495
Any blood products transfusion	2,434 (62.1)	389 (73.0)	2,045 (60.4)	< 0.001	1,710 (62.3)	724 (61.6)	0.646
RBC transfusion	2,010 (51.3)	321 (60.2)	1,689 (49.9)	< 0.001	1,414 (51.5)	596 (50.7)	0.618
Plasma transfusion	1,022 (26.1)	184 (34.5)	838 (24.7)	< 0.001	735 (26.8)	287 (24.4)	0.118
Platelet transfusion	1,821 (46.5)	318 (59.7)	1,503 (44.4)	< 0.001	1,281 (46.7)	540 (45.9)	0.653
Cryoprecipitate transfusion	545 (13.9)	142 (26.6)	403 (11.9)	< 0.001	382 (13.9)	163 (13.9)	0.956
Use of IABP (before extubation)	110 (2.8)	77 (14.4)	33 (1.0)	< 0.001	81 (3.0)	29 (2.5)	0.398
**Laboratory test**							
Preoperative NLR	1.8 (1.4, 2.5)	2.0 (1.5, 3.0)	1.8 (1.4, 2.5)	< 0.001	1.8 (1.4, 2.6)	1.8 (1.3, 2.5)	0.127
Preoperative PLR	103.7 (80.8,134.9)	104.4 (79.5, 142.9)	103.6 (81.0,134.2)	0.433	103.9 (81.0,136.0)	103.5 (79.8,133.3)	0.345
Preoperative LMR	4.2 (3.1, 5.5)	3.8 (2.6, 5.0)	4.3 (3.2, 5.5)	< 0.001	4.2 (3.1, 5.4)	4.2 (3.2, 5.5)	0.078
Hemoglobin (g/L)	130 (118, 140)	128 (115, 140)	130 (119, 140)	0.034	130 (118, 140)	129 (119, 140)	0.464
Anemia	1,435 (36.6)	236 (44.3)	1,199 (35.4)	< 0.001	1,003 (36.6)	432 (36.7)	0.920
Albumin (g/L)	40.5 (38.2, 42.8)	39.8 (37.5, 42.1)	40.6 (38.3, 42.9)	< 0.001	40.5 (38.2, 42.7)	40.5 (38.2, 42.8)	0.908
Albumin < 35 g/L	245 (6.3)	62 (11.6)	183 (5.4)	< 0.001	168 (6.1)	77 (6.5)	0.616
eGFR (mL/min/1.73m^2^)	95.2 (80.0,108.2)	86.7 (68.6, 100.4)	96.5 (81.8, 109.1)	< 0.001	94.7 (79.8, 108.3)	96.5 (80.1,107.9)	0.508
eGFR < 60 mL/min/1.73m^2^	276 (7.0)	88 (16.5)	188 (5.6)	< 0.001	185 (6.7)	91 (7.7)	0.265

BMI, body mass index; COPD, chronic obstructive pulmonary disease; CAD, coronary artery disease; MI, myocardial infarction; percutaneous coronary intervention; PCI; PAH, pulmonary arterial hypertension; EF, ejection fraction; CABG, coronary artery bypass grafting; CPB, cardiopulmonary bypass; ACC, aortic cross clamp; RBC, red blood cell; IABP, intra-aortic balloon pump; NLR, neutrophil–lymphocyte ratio; PLR, platelet–lymphocyte ratio; LMR, lymphocyte-to-monocyte ratio; eGFR, estimated glomerular filtration rate.

### Predictive model development

**Figure 2:**
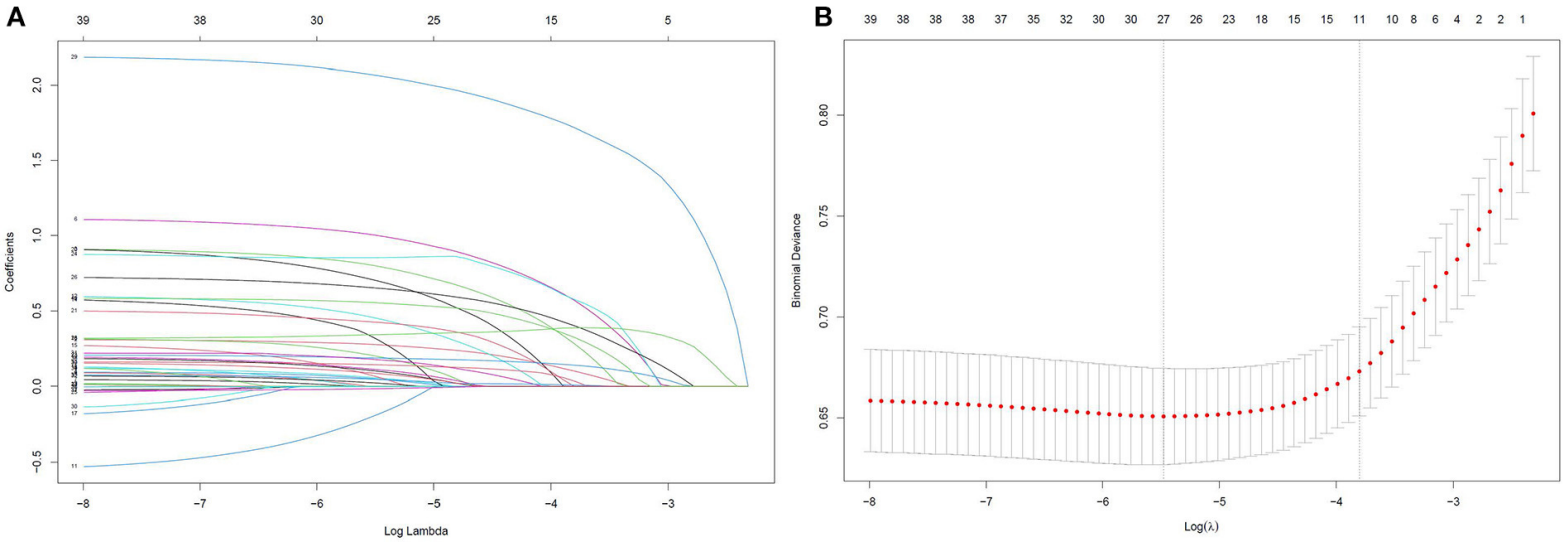
LASSO regression was used to select the potential risk factors. **(A)** LASSO coefficient curves for the 40 clinical variables; **(B)** Binomial Deviance of the LASSO model with different λ. The optimal value of λ (the vertical dashed line) for 10-fold cross-validation was used to select optimal variables. LASSO, least absolute shrinkage and selection operator.

Based on the training data, a total of 40 candidate parameters were entered into the LASSO regression analysis, and variables were selected according to the optimal lambda value values after the 10-fold cross-validation procedure (Figure 2). After the initial screening, 13 potential variables, including age, BMI ≥ 28 kg/m^2^, EF < 50%, history of cardiac surgery, use of vasoactive drugs, type of operation, emergency surgery, CPB ≥ 120 min, duration of surgery, IABP, eGFR < 60 mL/min/1.73 m^2^, intraoperative infusion of platelets and cryoprecipitate, were incorporated into the next multivariable logistic regression analysis. The results indicated that age, BMI ≥ 28 kg/m^2^, EF < 50%, history of cardiac surgery, type of operation, emergency surgery, CPB ≥ 120 min, duration of surgery, IABP and eGFR < 60 mL/min/1.73 m^2^ were independently associated with delayed extubation after cardiac surgery. Finally, these 10 predictors were introduced to construct a risk prediction model and assigned specific weight scores according to the corresponding coefficients (Table 2). The assigned scores for these predictors ranged from 1 to 13 and the possible total scores for each patient ranged from 0 to 64.

**Table 2:** Multivariable logistic regression model for predictors of delayed extubation and weighted score assignment.

**Predictors**	**Adjusted OR (95% CI)**	***P-*value**	**β coefficient**	**Weighted points**
Age (years)		< 0.001		
< 40			(reference)	
40–50	1.52 (0.90, 2.61)	0.12	0.419	2
50–60	1.75 (1.09, 2.89)	0.03	0.559	3
60–70	2.90 (1.80, 4.82)	< 0.001	1.065	6
≥70	4.69 (2.49, 8.89)	< 0.001	1.544	9
BMI ≥ 28 kg/m^2^	2.50 (1.72, 3.58)	< 0.001	0.915	5
EF < 50%	3.04 (2.09, 4.36)	< 0.001	1.111	6
History of cardiac surgery	2.41 (1.40, 4.05)	< 0.001	0.879	5
Type of operation		0.01		
CABG only			(reference)	
Valve only	1.19 (0.82, 1.76)	0.36	0.176	1
Aortic only	1.89 (1.04, 3.38)	0.03	0.635	4
CABG + valve	1.79 (1.15, 2.80)	0.01	0.584	3
Valve + aortic	1.95 (0.62, 5.54)	0.23	0.667	4
CABG + aortic	2.52 (0.92, 6.88)	0.07	0.925	5
Emergency surgery	2.38 (1.03, 5.46)	0.04	0.866	5
CPB ≥ 120 min	2.26 (1.67, 3.08)	< 0.001	0.817	5
Duration of surgery		< 0.001		
< 3 h			(reference)	
3–5 h	1.64 (0.92, 3.20)	0.12	0.497	3
5–7 h	1.62 (0.82, 3.39)	0.18	0.482	3
≥7 h	3.80 (1.71, 8.83)	< 0.001	1.335	8
Use of IABP (before extubation)	9.37 (5.43, 16,54)	< 0.001	2.238	13
Preoperative eGFR < 60 mL/min/1.73m^2^	1.94 (1.30, 2.88)	< 0.001	0.665	4

OR, Odds ratio; CI, confidence interval; BMI, body mass index; EF, ejection fraction; CABG, coronary artery bypass grafting; CPB, cardiopulmonary bypass; IABP, intra-aortic balloon pump; eGFR, estimated glomerular filtration rate.

### Predictive model validation

In this study, the median risk score was 10 in both cohorts with the highest risk score of 43 in the development cohort and 39 in validation cohort. Based on the distribution of total points, patients in the development and validation cohorts were classified into low- (0–8 points), medium- (9–12 points), and high-risk (≥13 points) groups. In both cohorts, there was significant difference for the proportion of patients with delayed extubation among the three risk groups. Compared with the low-risk patients, patients in medium-risk had ~2 times greater risk of delayed extubation, and patients in medium-risk had ~10 times greater risk (Table 3).

**Table 3:** Odds ratios of delayed extubation in the different risk groups.

	**Training set (*****N*** = **2,743)**			**Validation set (*****n*** = **1,176)**		
	***N* (%)**	**OR (95% CI)**	***P*-value**	***N* (%)**	**OR (95% CI)**	***P*-value**
Low-risk	26 (2.9%)	(reference)		18 (4.6%)	(reference)	
Medium-risk	82 (8.7%)	3.20 (2.07, 5.12)	< 0.001	34 (8.2%)	1.84 (1.04, 3.39)	0.042
High-risk	269 (29.7%)	14.15 (9.52, 21.92)	< 0.001	104 (28.3%)	8.22 (4.98, 14.31)	< 0.001

OR, odds ratio; CI, confidence interval.

**Figure 3:**
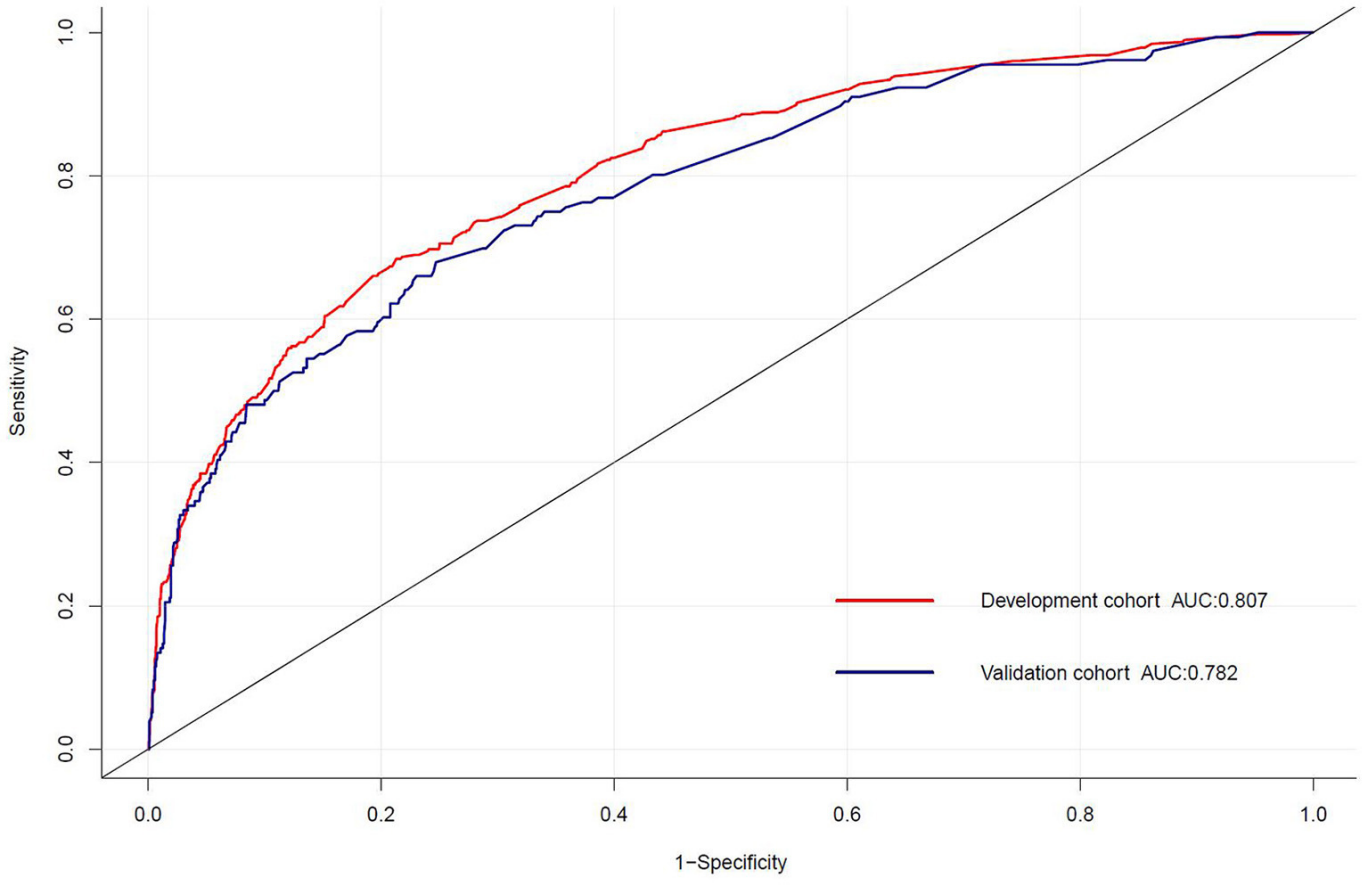
The receiver operating characteristic (ROC) curve for the prediction model of delayed extubation. The area under the curve (AUC) of the model exhibited good discriminating power in the development and validation cohorts.

**Figure 4:**
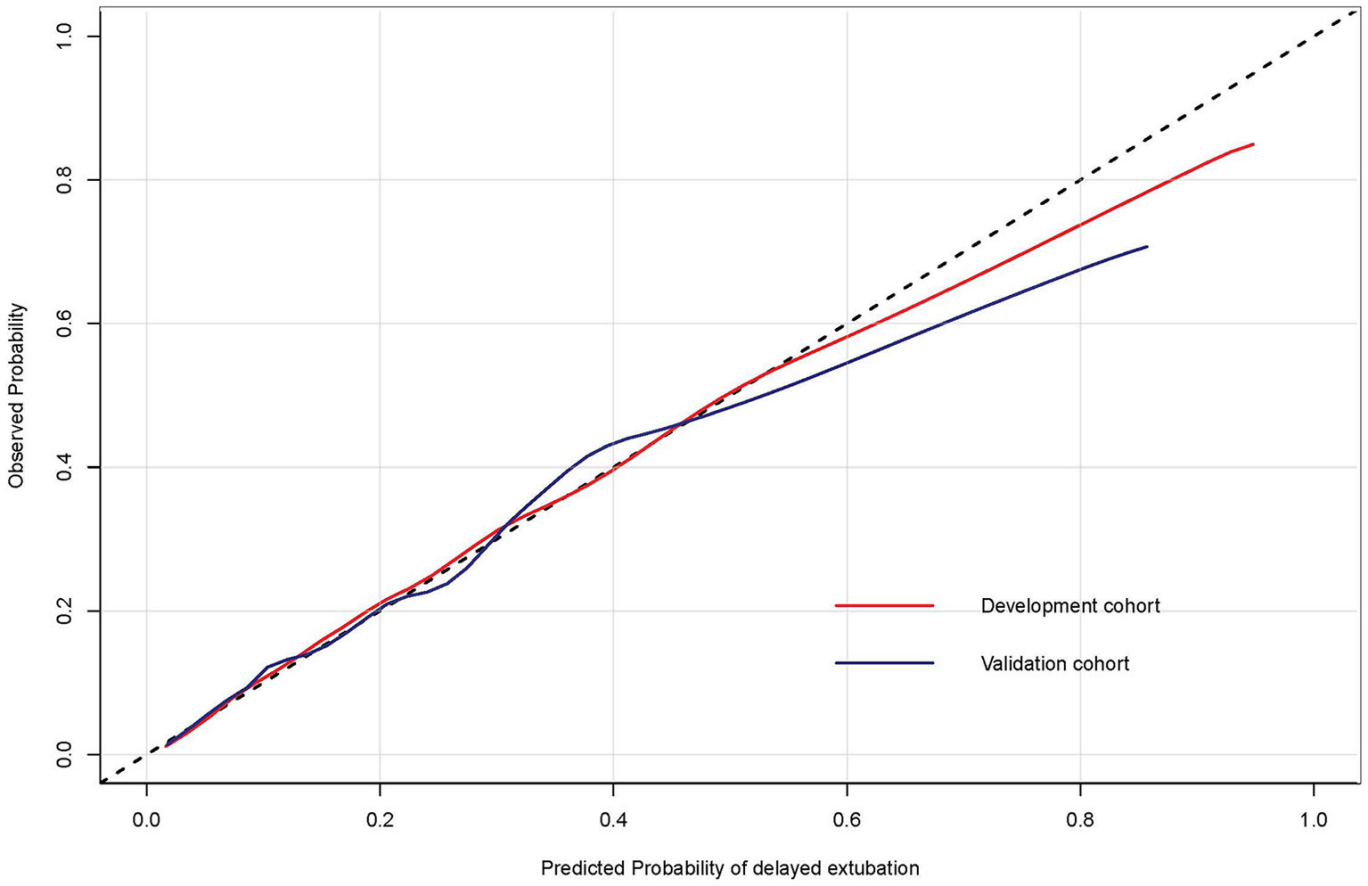
The calibration curves of the risk prediction model of delayed extubation. The diagonal dashed line indicated perfect prediction of the ideal model. The solid line represented the performance of the model in the development and validation cohorts, and being closer to the diagonal dashed line indicated that the model has better prediction ability.

For the predictive model, the reported area under the ROC is 0.807 (0.783, 0.832) in the development cohort and 0.782 (0.742, 0.823) in the validation cohort, indicating a good discrimination power (Figure 3). The overlapping of the actual and predicted calibration plots suggested good fit of the model (Figure 4). The calibration was further confirmed using the Hosmer-Lemeshow test, with a non-significant *P*-value of 0.401 in the development cohort and 0.901 in the validation cohort.

**Figure 5:**
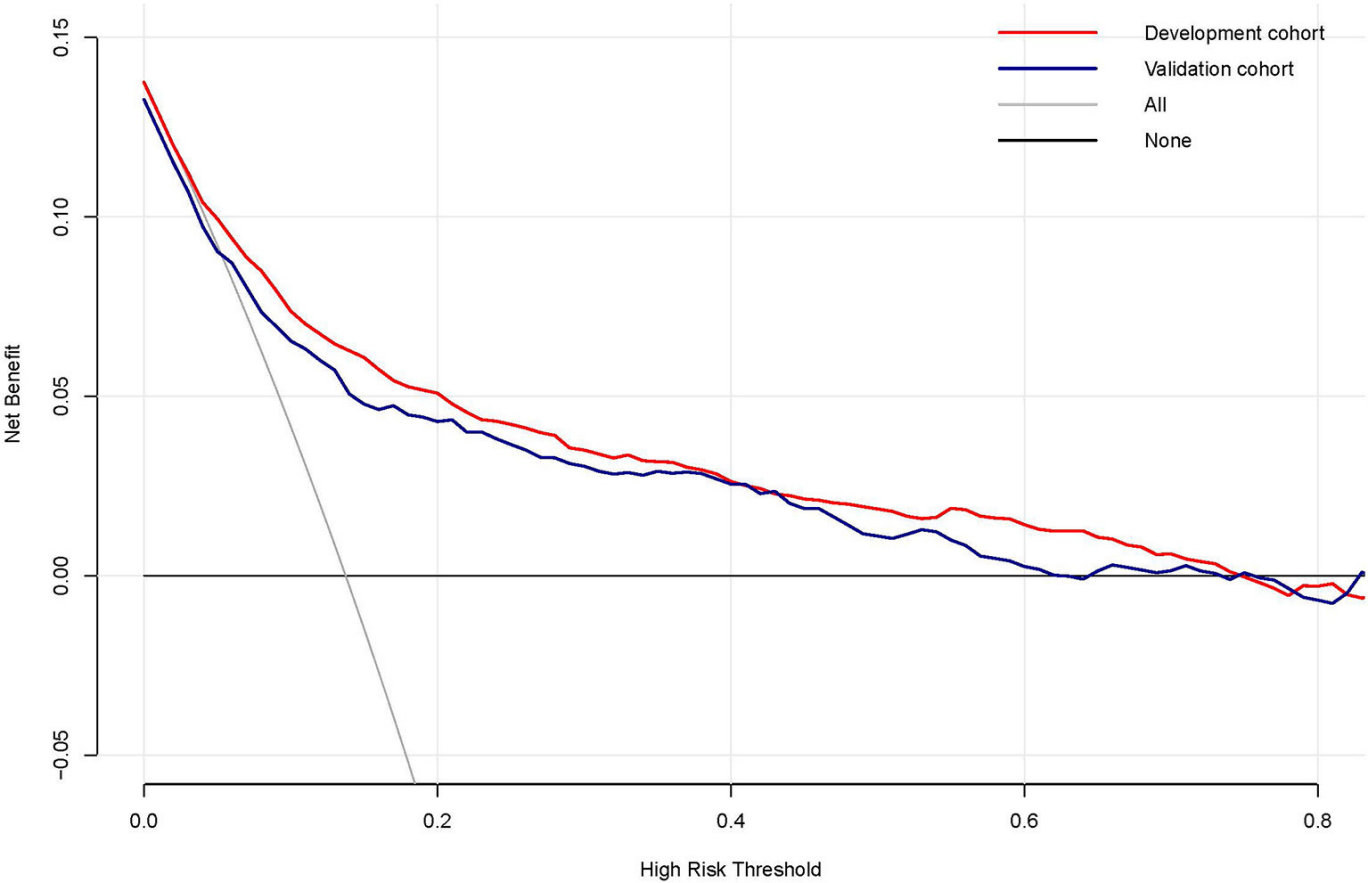
The decision curve analysis of the risk prediction model of delayed extubation. The vertical axis measured the net benefit. The gray solid line represented the assumption that no patient has delayed extubation. The black solid line represented the assumption that all patients appeared delayed extubation. The red line and blue line represented the prediction model from the development and validation cohorts, respectively.

**Figure 6:**
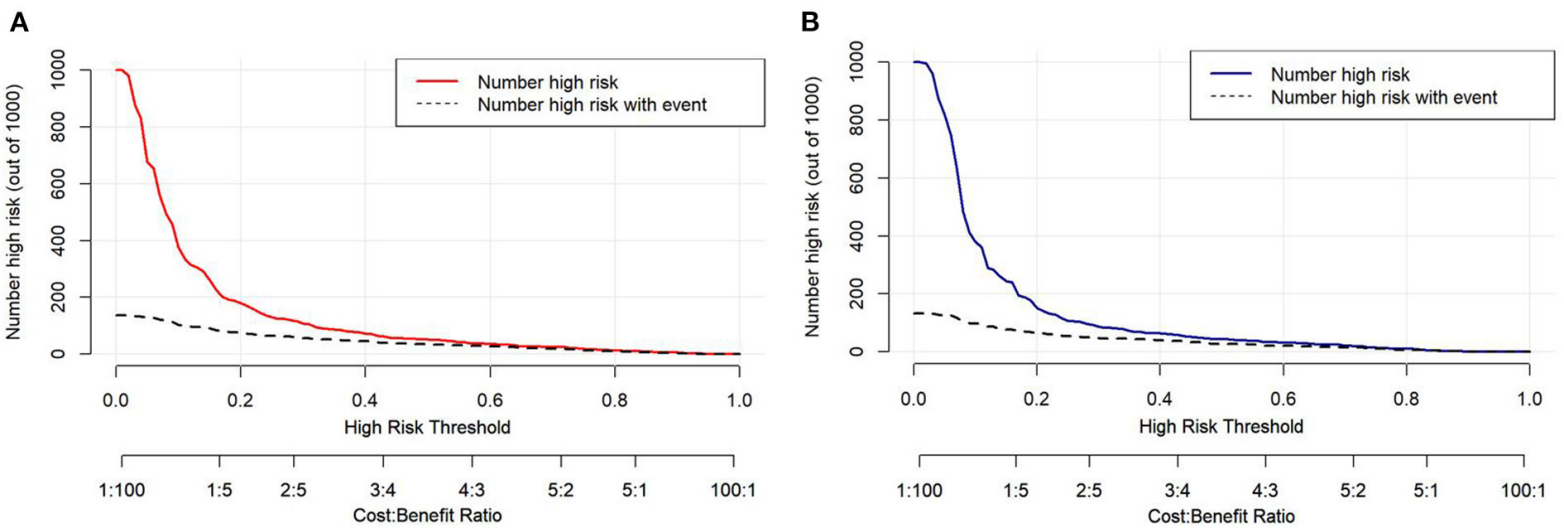
The clinical impact curves of the risk prediction model in the development cohort **(A)** and validation cohort **(B)**. The number of high-risk patients and the number of high-risk patients with delayed extubation were plotted at different threshold probabilities within a given population.

Decision curve analysis (DCA) was performed to explore the clinical usefulness of the model by quantifying the net benefits at different threshold probabilities. The probability threshold of the model was 3–74% in the development cohort and 3–62% in the validation cohort (Figure 5). Compared with the “all happened” and “none happened” assumptions, the prediction model could provide good predictive net benefit for a wide range of decision threshold. The clinical impact curves also suggested the higher usefulness of the prediction model in a realistic clinical setting (Figure 6).

### Clinical outcomes

To assess the potential impact of delayed extubation on patient prognosis, we compared several clinical outcomes including major postoperative complications and hospital length of stay (Table 4). Patients with delayed extubation had a median duration of postoperative mechanical ventilation of 68 (52, 92) h, compared with 21 (15, 16) h in patients without delayed extubation. Obviously, patients required delayed extubation tended to have a longer stay in the ICU and hospital than patients extubated within 48 h. Additionally, patients required delayed extubation in our study exhibited significantly higher extrapulmonary complications such as cardiac arrest, neurological complications and continuous renal replacement therapy (CRRT). Also, the significant differences of mortality in-hospital between two groups were observed (3.8 vs. 0.5%, *P* < 0.001).

**Table 4:** Comparison of clinical outcomes in patients with and without delayed extubation.

**Postoperative outcomes**	**Delayed extubation (*n* = 533)**	**Without delayed extubation** **(*n* = 3,386)**	***P*-value**
Mechanical ventilation (h)	68 (52, 92)	21 (18, 26)	< 0.001
ICU stay (d)	7 (5, 9)	3 (2, 4)	< 0.001
Length of hospital stay (d)	20 (15, 26)	14 (11, 17)	< 0.001
Pneumonia	241 (45.2)	439 (13.0)	< 0.001
Cardiac arrest	10 (1.9)	7 (0.2)	< 0.001
Neurological complications	12 (2.3)	16 (0.5)	< 0.001
CRRT	52 (9.8)	33 (1.0)	< 0.001
Re-admission to ICU	93 (17.4)	97 (2.9)	< 0.001
Re-intubation	59 (11.1)	56 (1.7)	< 0.001
Tracheotomy	39 (7.3)	21 (0.6)	< 0.001
Mortality In-hospital	20 (3.8)	18 (0.5)	< 0.001

ICU, intensive care unit; CRRT, continuous renal replacement therapy.

### Clinical risk assessment form

Considering the availability and practicality of the model, the final 10 predictors are formatted as a short and simple risk assessment form, which formally combines the risk score and risk stratification (Table 5). In addition to being reproduced for clinical use, the form provided is easy to document and preserve the risk assessment findings in clinical setting.

**Table 5:** Clinical risk assessment form for delayed extubation following cardiac surgery.

**Clinical risk assessment form**					
**Patient name:**		**Assessors:**		**Date of assessment:**	
Introductions: For each item below, place a tick (√) in the box that matches the information of the patient undergoing cardiac surgery					
1. Age: < 40	□ 0	5. Type of operation: CABG only	□ 0	8. Duration of surgery: < 3 h	□ 0
40–49	□ 2	Valve only	□ 1	3–5 h	□ 3
50–59	□ 3	Aortic only	□ 4	5–7 h	□ 3
60–69	□ 6	CABG + valve	□ 3	≥ 7 h	□ 8
≥ 70	□ 9	Valve + aortic	□ 4	9. Use of IABP	□ 13
2. BMI ≥ 28 kg/m^2^	□5	CABG + aortic	□ 5	10. Preoperative eGFR < 60mL/min/1.73m^2^	□ 4
3. EF < 50%	□ 6	6. Emergency surgery	□5	**Total points:**	
4. History of cardiac surgery	□ 5	7. CPB ≥ 120 min	□ 5		
**Identification of risk level:**	**□Low** (0–8 points)		**□Medium** (9–12 points)	**□High** (≥ 13 points)	

BMI, body mass index; EF, ejection fraction; CABG, coronary artery bypass grafting; CPB, cardiopulmonary bypass; IABP, intra-aortic balloon pump; eGFR, estimated glomerular filtration rate.

## Discussion

In this study, we identified 10 risk factors associated with delayed extubation: age, BMI ≥ 28 kg/m^2^, EF < 50%, history of cardiac surgery, type of operation, emergency surgery, CPB ≥ 120 min, duration of surgery, IABP and eGFR < 60 mL/min/1.73 m^2^. Then, we developed and internally validated the prediction model to generate individualized risk score of delayed extubation in cardiac surgery patients. The total risk score enables stratification of patients into low-risk, medium-risk and high-risk groups. Compared with the low-risk group ( ≤ 8 points), there was a significant trend toward increased incidence of delayed extubation in the medium-risk group (9–12 points) and high-risk group (≥13 points). The model was based on demographic and perioperative clinical variables for which information would be routinely obtained at the end of surgery. Consequently, this scoring model will contribute to an earlier assessment of extubation time and enable clinicians to develop specific treatment strategies as quickly as possible.

Duration of postoperative mechanical ventilation can serve as a meaningful end point for clinical trials, and successful ventilator weaning and extubation in the ICU is an important step of promoting postoperative recovery for all cardiac surgery patients. Furthermore, the poor prognosis of patients with delayed extubation also emphasized the importance of early identification in patients at risk. Although some risk factors in this model are similar to those reported in previous studies (8–11), our study differs from the perspective of statistical methods and has several advantages over previous conventional multivariable regression analysis. With the use of robust statistical modeling techniques, DCA and clinical impact curves, we present an effective, contemporary, yet simple predictive model of delayed extubation after cardiac surgery. As for the problem of multicollinearity between clinical variables, LASSO regression analysis in this study could minimize this problem and provide more accurate results. ROC curve, calibration plot and DCA and clinical impact curve were used for internal validation to ensure the stability and accuracy of this model.

Existing models of delayed extubation provide important findings about the risk factors associated with delayed extubation after cardiac surgery, including previous cardiac surgery, ejection fraction and CPB time (8–11). However, previous models lacked grade information on important variables such as age, type of surgery, and duration of surgery, which limited the generalizability and application value of these models. Moreover, until now, risk models able to risk stratify patients of delayed extubation after cardiac surgery appear to be less common in the literature. Risk stratification of predictive models is critical and helpful for clinicians to be more prepared and confident in the postoperative treatment of medium-risk and high-risk patients, as well as reasonable allocation of medical resources. In contrast, our model fills these gaps and highlights clinically relevant risk factors for delayed extubation, which greatly facilitates the clinician to better understand individual risk and decision-making.

Currently, there is no common threshold for the ideal extubation time after cardiac surgery, previous studies accepted very different time thresholds, ranging from 6 h to 21 days postoperatively (17). In the results of the analysis of the world literature, most investigators accepted a threshold of 24 or 48 h for extubation time to determine prolonged mechanical ventilation (18). Although the Society of Thoracic Surgeons (STS) defines prolonged mechanical ventilation as more than 24 h, this applies only to patient populations who underwent CABG alone. Considering that the study population of this study involved multiple types of cardiac surgery, we used a threshold of 48 h to define the delayed extubation. Due to different definitions and populations, the reported prevalence of delayed extubation varies widely in the literature, ranging from 6 to 12% (8–11). We divided the type and duration of surgery to determine its potential impact on delayed extubation. For this study, 13.6% of adult patients required delayed extubation for ventilatory support during ICU observation. The relatively high rates of delayed extubation may be related to the specific characteristics in our study population such as complex types of surgery. Patients undergoing more than two types of heart surgery tend to require longer duration of surgery and CPB time, and may be more unstable and at greater risk during surgery. In addition to longer mechanical ventilation and length of stay in the ICU, higher occurrence of major adverse cardiac or cerebrovascular events were also observed in patients with combined cardiac surgery (15, 19). We observed that nearly half of the patients undergoing aortic dissection surgery were mechanically ventilated for more than 48 h after surgery, with a similar incidence to previous reports in the literature (28.9–48.5%) (20–22). Our findings demonstrated that the risk of delayed extubation in patients undergoing aortic surgery was doubled compared to isolated CABG or valve surgery, which was similar to that of patients undergoing combined cardiac surgery.

In addition to common cardiac risk factors in previous models such as left ventricular ejection fraction and history of cardiac surgery, our model included patient-specific factors like age and BMI. Age-related changes in cardiac structure and function are well recognized (23), as are substantial changes in lung function (24). Risk of delayed extubation with each age group was explored by grouping patients by age. The results showed that the risk for delayed extubation after cardiac surgery increased significantly with age, especially after the age of 60 years. Reduced lung compliance and functional residual capacity are major causes of impaired lung function in obese patients, which is exacerbated by general anesthesia and mechanical ventilation (25). According to the Chinese guide (26), individuals with BMI ≥ 28 kg/m^2^ were considered obesity. Corresponding scores were assigned to each group to facilitate visualization of risks.

Activation of systemic inflammatory mediators induced by CPB increases the susceptibility to lung injury (16). Prolonged CPB time has repeatedly been reported as a risk factor for delayed extubation in the literature. As expected, in our model, the risk of delayed postoperative extubation increased ~2-fold when the duration of CPB exceeded 120 min. Hessels et al. (8) reported that Patients who received more than 210 min of CPB were nearly 4 times more likely to suffer delayed postoperative extubation. Obviously, the risk was related to the duration and complexity of the operation.

Patients undergoing emergency cardiac surgery are often in unstable condition and even life-threatening. Incomplete preoperative evaluation and preparation exposes patients to unknown risks, making it necessary for clinicians to carefully consider the risks before making extubation decisions. Meanwhile, the use of intra- or post-operative IABP means poorer cardiac function. Therefore, the need for longer ventilation times is usually a consequence of cardiopulmonary dysfunction or slower recovery after cardiac surgery.

Impaired preoperative renal function was classified as a presence eGFR < 60 mL/min/1.73 m^2^ (27). In the present study, patients with renal impairment had twice the risk of delayed extubation as those with normal renal function. Intraoperative exposure to CPB and concomitant use of multiple drugs further increase the total burden of renal metabolism (28), which may slow the excretion of drugs and their metabolites and early recovery from anesthesia.

Although these predictors reported here have clinical implications, there are some limitations to our work that require further study. First, all patients were from a single tertiary care center, which could limit the translation of our results to other centers. Second, due to the problem of missing data inherent in retrospective studies, a relatively small sample size was used in our study. Although our score model performed well in terms of discrimination and calibration, external populations are needed to determine whether our results are representative of those from other centers. Future prospective studies are needed to replicate our results and to determine the clinical implications of these retrospective findings. Third, detailed intraoperative data on mechanical ventilation, vasopressors and anesthetics were not included in our analysis.

## Conclusions

In this study, we observed that delayed extubation after cardiac surgery was associated with poorer postoperative recovery and higher risk of death. Using 10 easily obtainable and objective variables, we developed and validated a prediction score model to identify population at high risk for delayed extubation and stratify risk. We believe that this model will be a useful predictive tool to support informed clinical decision making and risk discussion.

## Data availability statement

The raw data supporting the conclusions of this article will be made available by the authors, without undue reservation.

## Ethics statement

The studies involving human participants were reviewed and approved by Ethics Committee of Union Hospital affiliated to Tongji Medical College of Huazhong University of Science and Technology. Written informed consent for participation was not required for this study in accordance with the national legislation and the institutional requirements.

## Author contributions

QW, WX, JL, and XL: study concept and design. YB and LC: acquisition and collection of data. XL and JL: analyzed the data. ZX, YW, JL, and XL: interpretation of data. XL: drafted the manuscript. QW and WX: manuscript revision and editing. All authors: final approval of manuscript.

## Funding

This study was supported by the National Key Research and Development Program of China (Grant No. 2018YFC2001903) and the National Natural Science Foundation of China (Grant Nos. 81873952 and 81901948).

## Conflict of interest

The authors declare that the research was conducted in the absence of any commercial or financial relationships that could be construed as a potential conflict of interest.

## Publisher's note

All claims expressed in this article are solely those of the authors and do not necessarily represent those of their affiliated organizations, or those of the publisher, the editors and the reviewers. Any product that may be evaluated in this article, or claim that may be made by its manufacturer, is not guaranteed or endorsed by the publisher.
